# Mesenteric desmoid tumor after robot-assisted laparoscopic cystectomy with bladder replacement: a case report

**DOI:** 10.1093/jscr/rjab529

**Published:** 2022-02-15

**Authors:** Sotaro Fukuhara, Masanori Yoshimitsu, Takuya Yano, Ichiya Chogahara, Rie Yamasaki, Shin Ebara, Masazumi Okajima

**Affiliations:** 1 Department of Surgery, Hiroshima City Hiroshima Citizens Hospital, 7-33, Motomachi, Naka-ku, Hiroshima 730-8518, Japan; 2 Department of Pathology, Hiroshima City Hiroshima Citizens Hospital, 7-33, Motomachi, Naka-ku, Hiroshima 730-8518, Japan; 3 Department of Urology, Hiroshima City Hiroshima Citizens Hospital, 7-33, Motomachi, Naka-ku, Hiroshima 730-8518, Japan

## Abstract

Desmoid tumors are a very rare disease associated with familial adenomatous polyposis, surgical trauma and hormonal factors. Surgical trauma is a critical trigger for sporadic desmoid tumors. Tumor development has been reported, and laparoscopic surgery has become more widely performed than the conventional open surgery. However, a few cases of desmoid tumors have developed after robot-assisted surgery. When desmoid tumors develop after cancer surgery, they are often difficult to distinguish from cancer recurrence. This differentiation is important for patients with bladder cancer because it helps determine the treatment plan. However, very few cases of mesenteric desmoid tumors after cystectomy for bladder cancer have been reported. Herein, we present a case of desmoid tumor that developed following robot-assisted laparoscopic cystectomy for bladder cancer. The tumor was resected via minilaparotomy with laparoscopic assistance for diagnostic treatment.

## INTRODUCTION

Desmoid tumors are very rare and account for ~3% of all soft tissue tumors [1]. Desmoid tumors do not metastasize but can be locally aggressive and have a high risk of local recurrence [1]. The development of desmoid tumors is associated with familial adenomatous polyposis, surgical trauma and hormonal factors [1, 2]. Approximately 30% of patients have a history of surgical intervention at the tumor site [3], and a few patients develop the tumors after robot-assisted surgery [4].

Very few cases of mesenteric desmoid tumors after cystectomy for bladder cancer have been reported [5, 6]. When abdominal desmoid tumors occur following cancer surgery, the differentiation between a desmoid tumor and cancer recurrence is often difficult [2, 5].

## CASE PRESENTATION

**Figure 1:**
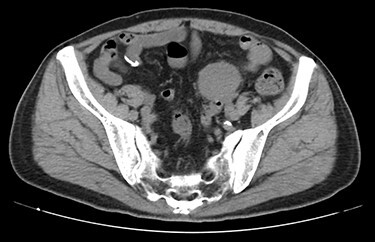
Contrast-enhanced CT showed a well-defined mass in contact with the small intestine that had heterogeneous enhancement.

**Figure 2:**
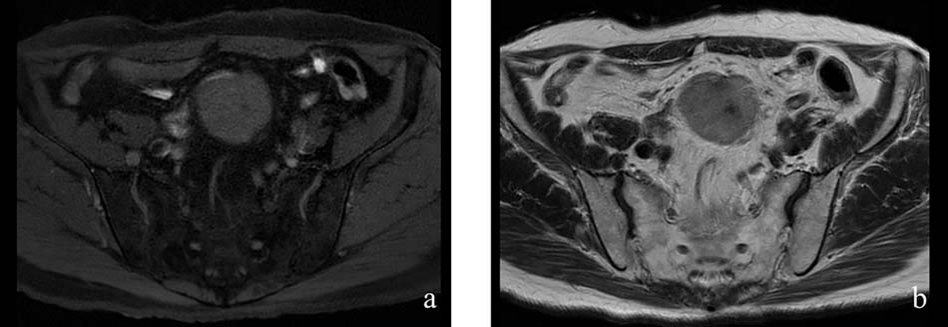
**Figure 2**: (**a**, **b**) The tumor had hypointensity on the T1-weighted images and mild hyperintensity on the T2-weighted images.

**Figure 3:**
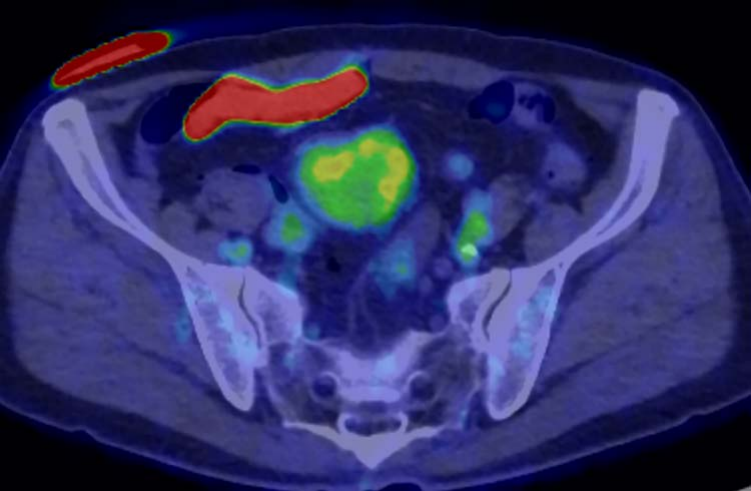
FDG-PET-CT showed FDG uptake in the tumor, with a SUVmax of 3.4.

A 70-year-old man underwent robot-assisted laparoscopic cystectomy with bladder replacement for bladder cancer. The pathological diagnosis was stage 0 (Tis, N0, M0). Nine months after the operation, no plain computed tomography (CT) findings indicated cancer recurrence. Thirteen months after the operation, contrast-enhanced CT showed a well-defined intra-abdominal mass, up to 50 × 40 mm in size, with heterogeneous enhancement. The tumor was in contact with the small intestine (Fig. 1). Magnetic resonance imaging (MRI) revealed an isolated mass with hypointensity on T1-weighted images and mild hyperintensity on T2-weighted images (Fig. 2a and b). Fluorine-18 fluorodeoxyglucose positron emission tomography CT (FDG-PET/CT) revealed mild FDG uptake in the tumor, with a maximum standardized uptake value (SUV max) of 3.4 (Fig. 3). Based on these findings, bladder cancer recurrence or mesenchymal tumors derived from the small intestine or mesentery were suspected. The tumor showed a rapid growth trend and required immediate therapeutic intervention. However, a differential diagnosis based on the imaging findings alone was difficult to achieve. Therefore, we decided to perform a diagnostic surgery to identify the intra-abdominal tumor.

**Figure 4:**
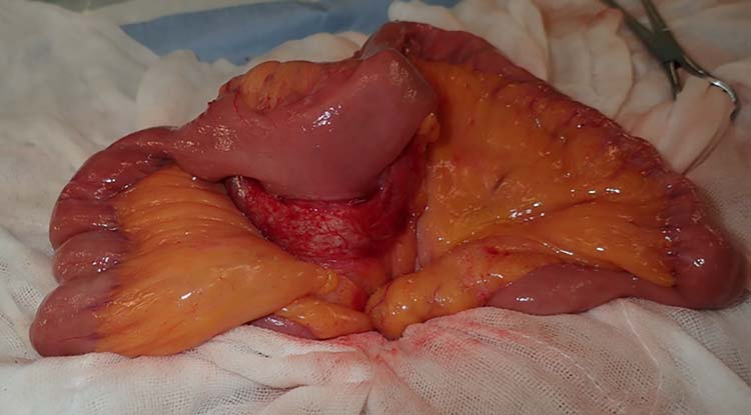
The tumor that developed from the mesentery was resected under a minilaparotomy.

**Figure 5:**
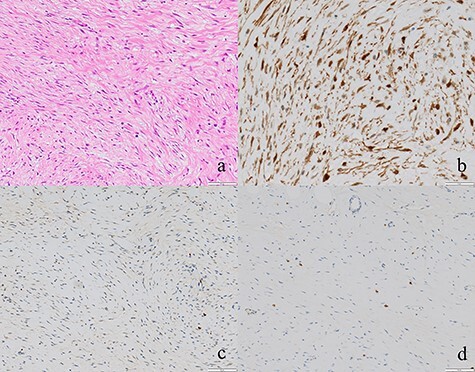
(**a**) Tumor cells comprised of fibroblasts were proliferating (×100). (**b**, **c**, **d**) The tumor cells were positive for β-catenin and negative for S-100 and c-kit (×50).

Regarding the intraoperative findings, we explored the abdominal cavity after minimally opening the upper abdomen. The abdominal wall extensively adhered to the small intestine and omentum in the lower abdomen. The adhesions made it difficult to locate the tumor on palpation. Therefore, we decided to identify the tumor using laparoscopy. A laparoscope was inserted from the place of the minilaparotomy wound with protector for pneumoperitoneum, and a 5-mm trocar was placed in the right abdomen. A hard mass could be visually recognized on the mesentery in the lower left abdomen using the laparoscope, and the mesentery was grasped with forceps. Adhesive detachment and abdominal wound extension were performed as much as necessary to remove the tumor from the abdominal cavity. The tumor was extracted under direct vision by partial resection of the small intestine (Fig. 4). The pathological findings revealed dense proliferation of spindle-shaped fibroblasts (Fig. 5a). Immunohistochemically, the tumor cells were positive for β-catenin and negative for S-100 and c-kit (Fig. 5b–d). Based on these findings, a diagnosis of desmoid-type fibromatosis was made. At the 12-month follow-up, the patient had no recurrence or metastases.

## DISCUSSION

Desmoid tumors can be found accidentally during postoperative follow-up of any cancer, and in such cases, the differentiation of desmoid tumors and cancer recurrence is often necessary. There have been reported two cases that developed after cystectomy for bladder cancer [5, 6]. CT showed that the tumor was a well-circumscribed homogeneous lesion with isodence or hyperdensity relative to the muscle [7]. On MRI findings, it often demonstrates hypointensity on T1-weighted images and heterogeneity on T2-weighted images [7]. However, these imaging findings are not specific to desmoid tumors. Mesenteric desmoid tumors can accumulate FDG uptake on PET-CT [2, 8]. Furthermore, FDG-PET/CT has been shown to be useful in assessing the response to treatment, but its role in assessing desmoid tumors remains unclear [8]. The above information underscores the difficulty of diagnosing desmoid tumors from imaging findings alone. In our case, cancer recurrence in the small intestine or mesentery was also differentiated. A prior study reported that, of bladder cancers with distant metastasis, only 3% were found in the intestine [9]. Thus, the small intestine or mesentery is not a predilection metastatic site of bladder cancer, and when a tumor develops from the small intestine mesentery and is found following surgery for bladder cancer, it may be necessary to immediately perform a diagnostic surgery.

Surgical excision is the standard treatment for patients with abdominal desmoid tumors. In recent years, initial observation, wait-and-see, has been recommended as a treatment policy for desmoid tumors [1, 10]. A retrospective study showed that ~50% of abdominal desmoid tumors had no tumor growth over a median follow-up of 38 months [1], and the spontaneous regression rate of abdominal desmoid tumors has been reported to be 33% [1, 10]. In our case, the tumor showed rapid growth over four months and had the possibility of bladder cancer recurrence. Therefore, tumor resection with diagnostic significance was appropriate.

In this case, the mesenteric desmoid tumor was probably caused by mechanical stimulation with surgical trauma due to robot-assisted laparoscopic cystectomy with bladder reconstruction. There are very few reports of desmoid tumors after robot-assisted surgery [4]. We assumed the existence of intra-abdominal adhesions due to previous surgery. The combined use of a laparoscope made it possible to easily identify the location of the tumor and the minimum surgical procedure necessary for adhesion detachment and wound extension. The abdominal cavity could be observed by laparoscopy, through the gap in the adhesion. The laparoscope is likely useful for reducing mechanical stimulation and finding the tumor easily and quickly during the operation, in cases with a surgical history. Desmoid tumors sometimes infiltrate surrounding tissues because they are locally aggressive [1], and a laparoscope can easily be used to evaluate tumor infiltration.

In conclusion, desmoid tumors that develop after bladder cancer surgery are difficult to distinguish from cancer recurrence and require diagnostic surgery. Diagnostic surgery may be needed to determine treatment strategy.

## References

[1] Burtenshaw SM , Cannell AJ, McAlister ED, Siddique S, Kandel R, Blackstein ME, et al. Toward observation as first-line management in abdominal desmoid tumors. Ann Surg Oncol 2016;23:2212–9. 27020588 10.1245/s10434-016-5159-6

[2] Yamamoto R , Mokuno Y, Matsubara H, Kaneko H, Iyomasa S. Multiple mesenteric desmoid tumors after gastrectomy for gastric cancer: a case report and literature review. Int J Surg Case Rep 2018;50:50–5. 30081320 10.1016/j.ijscr.2018.07.027 PMC6083430

[3] Lopez R , Kemalyan N, Moseley HS, Dennis D, Vetto RM. Problems in diagnosis and management of desmoid tumors. Am J Surg 1990;159:450–3. 2139764 10.1016/s0002-9610(05)81243-7

[4] Nakai M , Tazawa T, Wajima N, Muroya T, Mikami K, Hakamada K. A case of mesenteric fibromatosis after robot-assisted total gastrectomy for gastric cancer. Gan To Kagaku Ryoho 2016;43:1839–41. 28133149

[5] Zilberman DE , Mor Y, Fridman E, Ramon J. Mesenteric fibromatosis mimicking tumor recurrence following radical cystectomy and bladder replacement. Urol Case Rep 2015;3:40–1. 26793495 10.1016/j.eucr.2014.12.010 PMC4714273

[6] Pasciak RM , Kozlowski JM. Mesenteric desmoid tumor presenting as an abdominal mass following salvage cystectomy for invasive bladder cancer. J Urol 1987;138:145–6. 3599200 10.1016/s0022-5347(17)43026-6

[7] Hapgood C , DeLong A. Recurrent enlarging mesenteric desmoid tumor following remote surgical resection. Case Rep Radiol 2017;2017:2312617. 29403670 10.1155/2017/2312617 PMC5748304

[8] Makis W , Ciarallo A, Abikhzer G, Stern J, Laufer J. Desmoid tumour (aggressive fibromatosis) of the colon mimics malignancy on dual time-point 18F-FDG PET/CT imaging. Br J Radiol 2012;85:e37–40. 22308225 10.1259/bjr/43870228 PMC3473949

[9] Shinagare AB , Ramaiya NH, Jagannathan JP, Fennessy FM, Taplin ME, Van den Abbeele AD. Metastatic pattern of bladder cancer: correlation with the characteristics of the primary tumor. AJR Am J Roentgenol 2011;196:117–22. 21178055 10.2214/AJR.10.5036

[10] Bonvalot S , Ternès N, Fiore M, Bitsakou G, Colombo C, Honoré C, et al. Spontaneous regression of primary abdominal wall desmoid tumors: more common than previously thought. Ann Surg Oncol 2013;20:4096–102. 24052312 10.1245/s10434-013-3197-x

